# Physical morbidity and psychological and social comorbidities at five stages during pregnancy and after childbirth: a multicountry cross-sectional survey

**DOI:** 10.1136/bmjopen-2021-050287

**Published:** 2022-04-25

**Authors:** Mary McCauley, Sarah White, Sarah Bar-Zeev, Pamela Godia, Pratima Mittal, Shamsa Zafar, Nynke van den Broek

**Affiliations:** 1 Centre for Maternal and Newborn Health, Liverpool School of Tropical Medicine, Liverpool, UK; 2 Department of Obstetrics, Liverpool Women's NHS Foundation Trust,Crown street, Liverpool, UK; 3 Department of International Public Health, Liverpool School of Tropical Medicine, Liverpool, UK; 4 Department of Obstetrics, Centre for Maternal and Newborn Health, Lilongwe Office, Lilongwe, Malawi; 5 Centre for Maternal and Newborn Health, Nairobi Office, Nairobi, Kenya; 6 Department of Obstetrics and Gynecology, Vardhman Mahavir Medical College and Safdarjung Hospital, Delhi, India; 7 Fazaia Medical College, Air University, Islamabad, Pakistan

**Keywords:** public health, epidemiology, obstetrics

## Abstract

**Abstract:**

**Objective:**

Maternal morbidity affects millions of women, the burden of which is highest in low resource settings. We sought to explore when this ill-health occurs and is most significant.

**Settings:**

A descriptive observational cross-sectional study at primary and secondary-level healthcare facilities in India, Pakistan, Kenya and Malawi.

**Participants:**

Women attending for routine antenatal care, childbirth or postnatal care at the study healthcare facilities.

**Primary and secondary outcomes:**

Physical morbidity (infectious, medical, obstetrical), psychological and social comorbidity were assessed at five stages: first half of pregnancy (≤20 weeks), second half of pregnancy (>20 weeks), at birth (within 24 hours of childbirth), early postnatal (day 1–7) and late postnatal (week 2–12).

**Results:**

11 454 women were assessed: India (2099), Malawi (2923), Kenya (3145) and Pakistan (3287) with similar numbers assessed at each of the five assessment stages in each country. Infectious morbidity and anaemia are highest in the early postnatal stage (26.1% and 53.6%, respectively). For HIV, malaria and syphilis combined, prevalence was highest in the first half of pregnancy (10.0%). Hypertension, pre-eclampsia and urinary incontinence are most common in the second half of pregnancy (4.6%, 2.1% and 6.6%). Psychological (depression, thoughts of self-harm) and social morbidity (domestic violence, substance misuse) are significant at each stage but most commonly reported in the second half of pregnancy (26.4%, 17.6%, 40.3% and 5.9% respectively). Of all women assessed, maternal morbidity was highest in the second half of pregnancy (81.7%), then the early postnatal stage (80.5%). Across the four countries, maternal morbidity was highest in the second half of pregnancy in Kenya (73.8%) and Malawi (73.8%), and in the early postnatal stage in Pakistan (92.2%) and India (87.5%).

**Conclusions:**

Women have significant maternal morbidity across all stages of the continuum of pregnancy and childbirth, and especially in the second half of pregnancy and after childbirth.

**Strengths and limitations of this study**

- This is one of the first studies to comprehensively describe the burden of maternal multimorbidity at five assessment stages during pregnancy and after childbirth, using a standardised approach to assess the physical, psychological and social components of ill-health in combination with objective clinical and laboratory measurements.
- A large sample (11 454) of women across four low-income and middle-income countries were surveyed at five assessment stages during pregnancy and after childbirth.
- We describe when different types of maternal morbidity occur most frequently.
- The study population assessed women who had accessed care at a healthcare facility for routine antenatal care, childbirth or postnatal care, and did not assess the burden of maternal morbidity in women who did not access care.
- The study population we assessed in each country may not be generalisable to different regions of the same country or to other low-income and middle-income countries.

## Introduction

 The global maternal health agenda has undergone a refocus from preventing maternal deaths to promoting women’s health and wellness.^1 2^ Addressing the burden of maternal multimorbidity during and after pregnancy and the need to prevent and treat chronic and non-communicable diseases, is gaining importance as new focus for global initiatives, consistent with the current international strategy that all women have the right to the highest attainable standard of health and well-being, including all dimensions of health, that is, physical, psychological and social health.^1^,^3^ An international aim is to ensure that every woman in every setting has an equal chance to ‘survive and thrive’ during and after pregnancy and that every mother can enjoy a wanted and healthy pregnancy, safe childbirth and full recovery after childbirth.^1 2^ However, currently this is not the case for many women in low-income and middle-income countries (LMIC) with recent studies reporting that there is likely to be a significant burden of physical morbidity and comorbidities during and after pregnancy which may remain unrecognised and untreated if routine care services are not improved.^4^,^7^ Over the past 10 years, the focus of many maternal health intervention programmes in LMIC has been centred on care during childbirth and the time immediately after birth with the aim to reduce the global burden of maternal deaths, stillbirths and early neonatal deaths; ‘the triple return’.^2^ Focusing only on the number of women who die, ignores the women who suffer both severe and non-severe complications related to pregnancy and childbirth.^8 9^ Until recently, less emphasis has been placed on ensuring the availability and quality of maternity care during and after pregnancy, and the need to improve maternal health outcomes and experiences. Maternal morbidity represents a critical interface in the continuum between a healthy pregnancy and childbirth and maternal death.^10^ While some mothers will recover from their experienced ill-health with or without treatment, others will not. An increased awareness and understanding of the burden of physical morbidity and associated psychological and/or social comorbidity, along with prevention, early recognition and appropriate management where required, are important steps that need to be taken to improve maternal health, reduce adverse pregnancy outcomes and potentially avert preventable deaths.^4^,^11^ Maternal morbidity is defined as ‘any health condition attributed to and/or complicating pregnancy, and childbirth that has a negative impact on the woman’s well-being’.^12^ Several recent studies have attempted to describe and/or measure the burden of maternal morbidity in line with this new definition in women in low resources settings.^4^,^713^ However, there is still a lack of information regarding when (during pregnancy or after childbirth) this burden occurs and is most significant. This should inform when the optimal timing is for screening, prevention and management. The objective of this study was to assess the prevalence and types of maternal morbidity (infectious, medical/obstetrical) and psychological and social comorbidities for each of five different stages during and after pregnancy. Second, we explored and compared findings by geographical settings across four LMICs.

## Materials and methods

### Study design and settings

The details of the study design, setting, participants have been described previously.^6^ In summary, we conducted a descriptive observational cross-sectional study in India, Pakistan, Kenya and Malawi, across as representative sample of 12 secondary and 17 primary care level facilities in rural and urban areas.

### Participants

All women attending for antenatal care, childbirth or postnatal care at the study healthcare facilities were eligible for inclusion. Women who were too ill and unable to speak to participate (eg, altered conscious level after an eclamptic seizure) were excluded. Each woman was assessed at one of the five stages of pregnancy: first half of pregnancy (≤20 weeks), second half of pregnancy (>20 weeks), birth (within 24 hours of childbirth), early postnatal (days 1–7) and late postnatal (weeks 2–12). For the antenatal assessments, gestation was calculated based on the women’s last menstrual period or the results of a dating scan if available. Women were recruited sequentially until the target sample size for each assessment stage was reached in each healthcare facility. All women who consented to take part in the study were interviewed and had a full clinical examination, and basic urine and serological investigations performed by trained healthcare providers (midwives and doctors). Data were collected using a standardised structured questionnaire formatted onto electronic tablets in India, Pakistan and Kenya. Paper questionnaires were used in Malawi. Demographics including age, marital status, occupation and educational level and socioeconomic status were assessed. Current physical symptoms were assessed using 76 questions covering six organ systems—cardiopulmonary, gastrointestinal, musculoskeletal, urogynaecology, obstetric and breast, and miscellaneous (dermatology, endocrine, neurological, immunology, ear–nose–throat). Psychological health was assessed using the Edinburgh Postnatal Depression Scale (EPDS) using a cut-off point of >10.^15^ The ‘Hurt, Insulted, Threatened, Screamed at’ (HITS) questionnaire was used to assess domestic violence, from the husband or partner and/or other family members (with a score of >10 indicating significant abuse).^16^ Four questions from the ‘Alcohol, Smoking and Substance Involvement Screening Test (ASSIST)’ questionnaire were included.^17^ Clinical parameters including height, weight, pulse rate (PR), respiratory rate (RR), blood pressure (BP) and oral temperature (T) were measured and the conjunctiva, sclera, breast, abdomen (general and obstetrical) were examined. Inspection of the perineum and/or speculum examination was only conducted if clinically indicated (for symptoms of vaginal discharge, bleeding, pain). Urinalysis was performed using Multistix GP. A simple finger prick test was used to obtain one capillary (<0.5 mL) of blood for use in four rapid diagnostic tests: haemoglobin (HemoCue), malaria (Humasis), syphilis and HIV (SD BIOLINE HIV/Syphilis Duo) and C-reaction protein (CRP) (QuickRead). A reported symptom of cough >two weeks was used to identify chest infection and/or suspected tuberculosis. Anaemia was classified as haemoglobin less than 110 g/L.^18^ Hypertension was classified as BP ≥140/90.^19^ Pre-eclampsia was defined as BP ≥140/90, and proteinuria (PR >++ on urinalysis) after 20 weeks’ gestation.^19^ We amended the Systematic Inflammatory Response Syndrome (SIRS) score to define possible early infection as the presence of two or more of the following: (1) T>38°C or <36°C, (2) PR >90 beats per min, (3) RR >20 breaths per min or (4) raised CRP (defined as >5 mg/dL at each assessment stage, apart from the early postnatal period (first 7 days) where raised CRP was defined as >10 mg/dL.^20^ Antenatal haemorrhage was defined as women who reported bleeding per vagina during pregnancy and/or who had this confirmed on examination. Incontinence was defined as all women who reported any leakage of urine and/or had this confirmed-on examination. Summative physical morbidity was categorised as (1) infectious or (2) medical or obstetrical. Infectious morbidity included: HIV, malaria, syphilis, chest infection and/or suspected tuberculosis and a SIRS score of ≥2. Medical or obstetrical morbidity included: anaemia, hypertension, pre-eclampsia, antenatal haemorrhage and urinary incontinence. Psychological morbidity was defined as an EPDS score of ≥10 and/or thoughts of self-harm. Social morbidity was defined as a woman reporting any domestic violence (HITS score >4) and/or any substance misuse. Maternal morbidity was considered as at least one physical morbidity, or psychological or social comorbidity.

### Sample size calculation and statistical analysis

In Pakistan, Kenya and Malawi for each of the five assessment stages, data were collected for a minimum of 576 women across two levels of healthcare facility (primary and secondary) selected by stratified cluster sampling.^6^ In India, as the study was conducted in one facility (secondary level facility offering primary and secondary care) a cluster sampling approach was not required, giving an amended sample size of 1900 with a minimum of 380 women per assessment stage.^6^ This sample size had 95% power to detect the presence of any morbidity with a prevalence greater than 1%. Data analysis was performed using SPSS V.22 and Stata V.12.1. Unless otherwise stated, all percentages reported use the total sample size for the relevant country. Where a substantial percentage of women have data missing for a variable (>10%) this is stated in the text, but the numbers missing are not tabulated. Percentages are derived using the number of women who responded per assessment stage. Analysis was conducted separately for each stage of pregnancy for each country and then for each stage of pregnancy for women in all countries combined as a cohort.

### Patient and public involvement

No patient nor members of the public were involved in the design, conduct or dissemination of this study.Results

### Study population

A total of 11 454 women across four LMICs were assessed: India (2099), Pakistan (3287), Kenya (3145) and Malawi (2923) with similar numbers of women assessed at each of the five stages, during and after pregnancy. The sociodemographic and obstetrical characteristics of women per stage are displayed in online supplemental table 1. Results are reported for the combined cohort of women and where there are significant differences between the women from each country, these are commented on in the narrative text. Data obtained for each country for each pregnancy stage are detailed in online supplemental tables 2–9.

### Reported symptoms and severity for each stage of pregnancy

**Figure 1:**
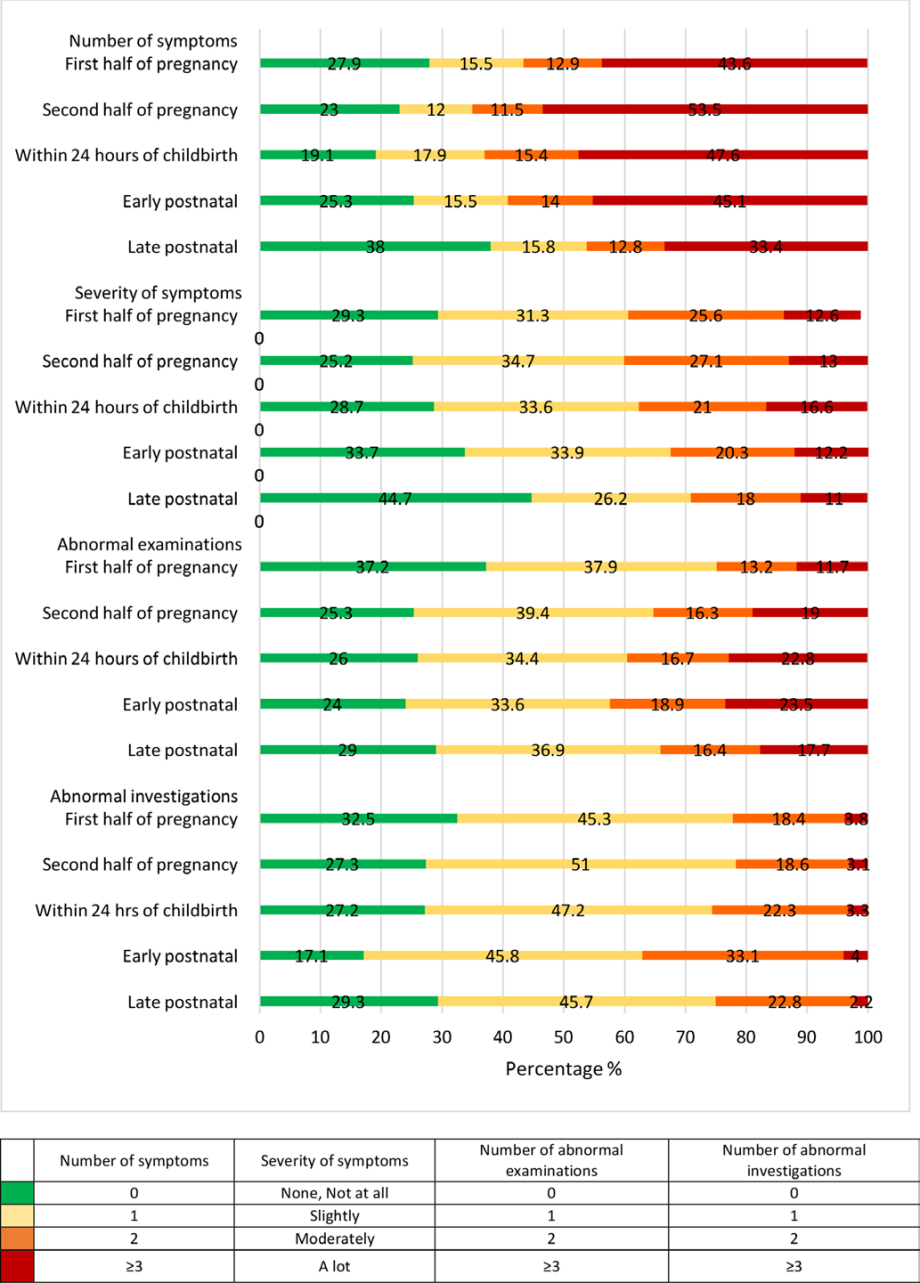
Histogram of number and severity of symptoms reported, number of abnormal clinical examinations, number of abnormal investigations per stage per combined study population (n=11 454).

Overall, 8420 women (73.5%) reported at least one physical symptom of ill-health at any of the assessment points during or after pregnancy with a median (IQR) of 2 (0–5) symptoms per woman. The number of symptoms reported by women varied statistically significantly with stage of pregnancy (using categories presented in figure 1, χ^2^=330, df=12, p<0.001). In the second half of pregnancy the median (IQR) was 3 (1-6), significantly higher than for the other stages. In the late postnatal stage it was 1 (0–3); whereas for the first half of pregnancy it was 2 (0–4), within 24 hours of childbirth it was 2 (1–5) and early postnatal it was 2 (0–5). The percentage of women with reported physical ill-health (at least one symptom reported) was highest within 24 hours of childbirth (80.9%) and the percentage of women who reported at least four symptoms was highest (43.1%) in the second half of pregnancy (figure 1). The percentage of women reporting that they had symptoms that were bothering them ‘a lot’ was similar at each stage of pregnancy and ranged from 11.0% to 16.6% (figure 1). Women living in Pakistan reported higher severities of symptoms compared with India, Kenya and Malawi; and across all five assessment points. At the start of the pregnancy 11.7% of women had ≥3 abnormalities on clinical examination and 22.2% of women had ≥2 abnormalities on urine and/or blood investigation (figure 1). The highest percentage of women with ≥3 abnormalities on clinical examination or ≥2 abnormalities on urine and/or blood investigation were in the early postnatal stage (23.5% and 37.1%, respectively) (figure 1).

### Infectious morbidity

The percentage of women with early signs of infection (SIRS score ≥2) was highest in the early postnatal period (26.1%) and second half of pregnancy (25.1%). Similar percentages of women were detected to be HIV positive across all five assessment stages. The percentage of women with malaria and syphilis was highest in the first half of pregnancy (3.9% and 1.6%, respectively) with decreased prevalence at subsequent points (table 1). For the three main diseases combined (malaria, syphilis and HIV), prevalence was 8.4% overall and highest in the first half of pregnancy (10.0%). This varied per country (online supplemental tables 2–5) and was 0.4% in India, 0.6% in Pakistan with no syphilis and rare cases of malaria), 4.1% in Kenya (mainly HIV) substantially higher in Malawi (27.9% including HIV 14.3%, malaria 10.2% and syphilis 3.4%). The percentage of women with a positive SIRS score was highest in Kenya in the early postnatal period (44.8%) followed by Malawi at 30.1% in the early postnatal period, 16.6% in India and 15.9% in Pakistan, both in the second half of pregnancy (online supplemental tables 2–5).

**Table 1:** Physical multimorbidity (infectious, medical, obstetrical) per assessment stage for all countries combined (n=11 454)

Assessment stage	First half of pregnancy	Second half of pregnancy	Within 24 hours of childbirth	Early postnatal	Late postnatal	Total
Number of women*	2204	2425	2250	2264	2311	11 454
	**n (%**)	**n (%**)	**n (%**)	**n (%**)	**n (%**)	**n (%**)
Infectious morbidity						
HIV	99 (4.5)	107 (4.4)	107 (4.8)	123 (5.4)	116 (5.0)	551 (4.8)
Malaria	85 (3.9)	73 (3.0)	59 (2.6)	43 (1.9)	49 (2.1)	309 (2.7)
Syphilis	36 (1.6)	24 (1.0)	19 (0.8)	16 (0.7)	12 (0.5)	107 (0.9)
Positive screening for chest infection/ possible tuberculosis	18 (0.8)	24 (1.0)	10 (0.4)	12 (0.5)	10 (0.4)	74 (0.6)
Septic Inflammatory Response Syndrome (SIRS)†	480 (21.8)	609 (25.1)	472 (21.0)	590 (26.1)	492 (21.3)	2643 (23.1)
Medical or obstetrical morbidity						
Anaemia‡	900 (40.8)	1226 (50.6)	1096 (48.7)	1213 (53.6)	985 (42.6)	5420 (47.3)
Severe anaemia	28 (1.3)	47 (1.9)	41 (1.8)	63 (2.8)	23 (1.0)	202 (1.8)
Body mass index ≤18.5 kg/m^2^	66 (3.0)	61 (2.5)	34 (1.5)	68 (3.0)	58 (2.5)	287 (2.5)
Body mass index >30 kg/m^2^	786 (35.6)	1264 (50.2)	912 (40.5)	665 (29.3)	761 (32.9)	4867 (42.5)
Hypertension	33 (1.5)	111 (4.6)	82 (3.6)	66 (2.9)	48 (2.1)	340 (3.0)
Pre-eclampsia	n/a	51 (2.1)	18 (0.8)	17 (0.7)	n/a	86 (1.2)
Urine incontinence	67 (3.0)	161 (6.6)	69 (3.1)	44 (1.9)	76 (3.3)	417 (3.6)
Antenatal haemorrhage	139 (6.3)	74 (3.0)	n/a	n/a	n/a	213 (4.6)
At least one medical or obstetrical condition	998 (45.3)	1328 (54.8)	1135 (50.4)	1245 (55.0)	1017 (44.0)	5723 (50.0)

* Where data were missing for a condition, the condition was regarding as being absent for purposes of deriving morbidities; % missing was: HIV 9.7%, malaria 5.3%, syphilis 8.9%, screening for chest infection/tuberculosis 2.0%, nutritional status 2.9%, anaemia 1.9%, blood pressure 2.3%, urine incontinence 0.5%.

† CRP was not measured at some primary level facilities in Malawi and Pakistan. Only participants for whom a CRP result was obtained are included in these statistics.

‡ Anaemia is defined as haemoglobin <110 g/L and severe anaemia is defined as haemoglobin <70 g/L.

CRP, C-reaction protein.

### Medical and obstetrical morbidity

Overall, one in two women had anaemia during or after pregnancy, the prevalence was highest in the early postnatal period (53.6% with 2.8% severe anaemia) with 40% of women anaemic in early pregnancy (table 1). The prevalence of anaemia was particularly high in Pakistan in the second half of pregnancy (76.8% with 3.5% severe anaemia), and in India in the early postnatal period (71.5% with 3.9% severe anaemia) (online supplemental tables 2–5). With regards to nutritional status, 3.0% of women had a low body mass index (<18.5 kg/m^2^) in the first half of pregnancy and in the early postnatal stage (table 1). Overall, the highest prevalence of hypertension or pre-eclampsia was in the second half of pregnancy (4.6% and 2.1%, respectively), and this was similar across all four countries; except in India, where the highest percentage of women with pre-eclampsia was in the early postnatal stage (0.2%). Urinary incontinence was consistently most commonly reported in the second half of pregnancy (6.6% overall) dropping to 3.3% in the late postnatal period. Urinary incontinence was comparatively higher in Pakistan with late postnatal urinary incontinence in 10.2% of women in the late postnatal period compared with nil in India, 0.3% in Malawi and 1.4% in Kenya (online supplemental tables 2–5). Overall, antenatal haemorrhage was reported in 6.3% of women in the first half of pregnancy stage and 3.0% in the second half of pregnancy. Overall, as a combined cohort 50.0% of women had at least one medical or obstetrical morbidity; with the highest prevalence in the early postnatal stage in India (72.0%) and Kenya (34.5%) or the second half of pregnancy in Pakistan (80.6%) and Malawi (44.8%) (online supplemental tables 2–5).

### Psychological comorbidity

Overall, 26.7% of women reported psychological morbidity; 22.9% of women had an EPDS score of ≥10; and 15.2% of women reported thoughts of self-harm.^6^ Depression was the the most common form of psychological morbidity at each assessment stage, with the highest percentage of women reporting symptoms of depression or thoughts of self-harm in the second half of pregnancy (26.4% and 17.6%, respectively) compared with the early (22.2% and 15.5%) or late postnatal periods (20.3% and 15.7%) (table 2). These trends were similar in India, Pakistan and Kenya but in Malawi, psychological morbidity was highest within 24 hours of childbirth (18.9%) (online supplemental tables 6–9).

**Table 2:** Psychological and social comorbidities of women per assessment stage for all countries combined (n=11 454)

Assessment stage		First half of pregnancy	Second half of pregnancy	Within 24 hours of childbirth	Early postnatal	Late postnatal	Total
Number of women*		2204	2425	2250	2264	2311	11 454
		**n (%**)	**n (%**)	**n (%**)	**n (%**)	**n (%**)	**n (%**)
Psychological morbidity							
EDPS ≥10		494 (22.4)	641 (26.4)	511 (22.7)	503 (22.2)	469 (20.3)	2618 (22.9)
Thoughts of self-harm		246 (11.2)	426 (17.6)	359 (16.0)	351 (15.5)	362 (15.7)	1744 (15.2)
EDPS ≥10 and/or thoughts of self-harm		542 (24.6)	721 (29.7)	630 (28.0)	591 (26.1)	575 (24.9)	3059 (26.7)
Social morbidity							
Domestic violence							
HITS score >4	Husband and/or family	657 (29.8)	978 (40.3)	756 (33.6)	770 (34.0)	722 (31.2)	3883 (33.9)
	Husband	475 (21.5)	776 (32.0)	597 (26.5)	602 (26.6)	560 (24.2)	3010 (26.3)
	Family	328 (14.9)	444 (18.3)	358 (15.9)	341 (15.1)	334 (14.4)	1805 (15.8)
HITS score >10	Husband and/or family	161 (7.3)	276 (11.4)	172 (7.6)	187 (8.3)	170 (7.4)	966 (8.4)
	Husband	101 (4.6)	223 (9.2)	109 (4.8)	127 (5.6)	121 (5.2)	681 (6.0)
	Family	74 (3.4)	80 (3.3)	85 (3.8)	79 (3.5)	73 (3.2)	391 (3.4)
Substance misuse							
Use of alcohol, sedatives, inhalants, tobacco in last 3 months		135 (6.1)	112 (4.6)	122 (5.4)	146 (6.5)	157 (6.8)	672 (5.9)
Intervention required (ASSIST score >4)		38 (1.7)	38 (1.6)	34 (1.5)	42 (1.9)	20 (2.2)	202 (1.8)

ASSIST, Alcohol, Smoking and Substance Involvement Screening Test; EPDS, Edinburgh Postnatal Depression Scale; HITS, Hurt, Insulted, Threatened, Screamed at.

### Social comorbidity

Overall, 3883 (33.9%) women reported domestic violence (HITS >4) and 969 (8.5%) reported higher levels of domestic violence (HITS >10) from their partner/husband and or family members.^6^ The highest percentage of women who reported domestic violence (HITS >4), and higher levels of domestic violence (HITS >10) was in the second half of pregnancy stage (40.3% and 28.6%, respectively) (table 2). Higher percentages of women reported domestic violence in the second half of pregnancy in Pakistan (60.4%), Kenya (29.4%) and Malawi (24.3%), compared with India, where more women reported domestic violence in the early postnatal period (43.7%) (online supplemental tables 6–9).

Overall, 672 (5.9%) women reported substance misuse, of which 202 (1.8%) required intervention and this was highest in Pakistan and India in the early postnatal period (12.0% and 2.8%, respectively) (online supplemental tables 6 and 7) and in the late postnatal period in Kenya (9.6%) or within 24 hours of childbirth in Malawi (5.3%) (online supplemental tables 8 and 9).

### Multimorbidity for each pregnancy stage

**Figure 2:**
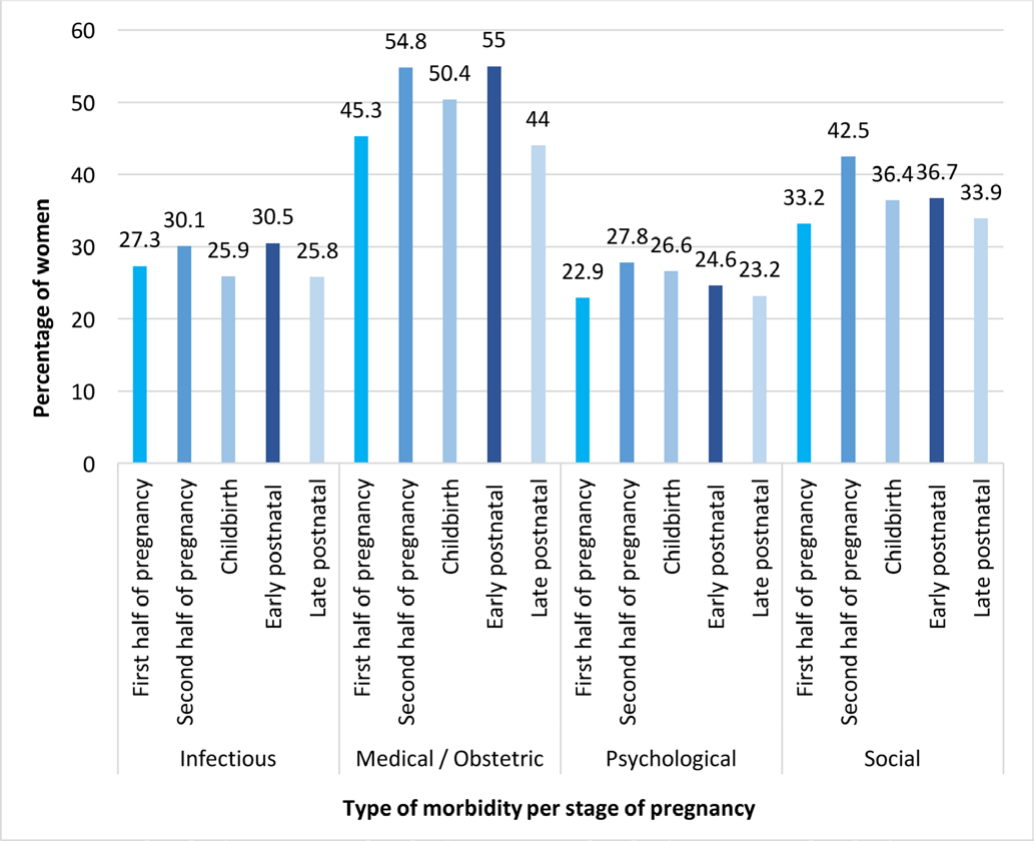
Percentage of women with infectious, medical/obstetrical, psychological and social morbidity per stage per combined study population (n=11 454).

Overall, 8936 (78.0%) women reported maternal morbidity (infectious, medical, obstetrical, psychological or social).^6^ The percentages of women reporting maternal morbidity were Pakistan: 90.1%, India: 83.9%, Malawi: 71.9% and Kenya: 67.1%. Of all women assessed, occurrence of maternal morbidity was highest in the second half of pregnancy (81.7%); followed by the early postnatal stage (80.5%), and within 24 hours of childbirth (79.0%). Similar percentages of women had maternal morbidity in the first half of pregnancy (74.8%) and the late postnatal stage (73.9%). The highest occurrence of maternal morbidity was in the second half of pregnancy in Kenya (73.8%) and Malawi (73.8%). In India and Pakistan, the highest percentage of women who had maternal morbidity was in the early postnatal stage (87.5% and 92.2%, respectively). As a combined cohort, the different types of maternal morbidity occurred in similar measures across the continuum of pregnancy and childbirth (figure 2). The range of variation in the means was about 5% for infectious and psychological morbidity, and about 10% for medical/obstetrical and social morbidities (figure 2).

## Discussion

### Main findings

Women have significant maternal morbidity during pregnancy and after childbirth. Prevalence of maternal morbidity is highest in the second half of pregnancy and after childbirth. Most women report physical symptoms after childbirth (80.9%), and the maximum number of different symptoms is experienced in the second half of pregnancy with a median (IQR) of 3 (1–6) symptoms reported per woman. In this population of women attending for routine antenatal or postnatal care, the percentage of women with an abnormal examination and/or investigation was highest in the first half of pregnancy (62.8% and 67.3%, respectively). The burden of infectious morbidity was highest in the early postnatal stage with 26.1% of women having possible early signs of infection (SIRS score ≥2). However, early signs of infection also occurred in 1 in 5 women early or late in pregnancy and in the late postnatal period. The burden of infectious disease varied per country with malaria, syphilis and HIV more common in Malawi and Kenya (27.9% and 4.1%, respectively) than in Pakistan or India although up to 1 in 15 women have evidence of infective morbidity most commonly in the second half of pregnancy (14.6% in India and 12.6% in Pakistan). Anaemia including severe anaemia is most commonly seen in the early postnatal stage (53.6%) but up to 64.1% of women in Pakistan are already anaemic in the first half of pregnancy. As expected, hypertension, pre-eclampsia and urinary incontinence were more common in the second half of pregnancy and haemorrhage more common in early pregnancy. Psychological morbidity including symptoms of depression and thoughts of self-harm (29.7% and 17.6%, respectively) was most commonly diagnosed in late pregnancy with still significant but lower prevalence in the late postnatal period (24.9% and 15.7%). Similarly, social morbidity was found to be highest in the second half of pregnancy with overall 40% of women reporting domestic violence from their husband or family. Substance abuse was comparatively low with up to 2.2% of women overall having an ASSIST score of >4 in the late postnatal period.

### Strengths and limitations of the study

To the best of our knowledge, this is the first study to measure the burden of maternal morbidity in a large sample (11 454) of women across four LMICs considering five separate stages during pregnancy and after childbirth. Our study provides measurements of maternal physical morbidity (infective, medical or obstetrical) as well as comorbidities (psychological and/or social) using clear and concise definitions and clinical assessment methodology, enabling comparisons between different settings and countries. We documented very low refusal rate (range 1.1%–2.5%) and working in ‘real life ‘settings during routine antenatal and postnatal care provision in each setting and conclude that it is acceptable to women and their healthcare providers, and in principle feasible, to screen women for all the different types of maternal morbidity as part of routine healthcare consultations. Further strengths of this study are that both subjective (self-reported symptoms) and objective measures (examination and investigations) are included enabling a ‘diagnosis’ to be made where needed rather than using a syndromic approach only. Finally, women were assessed as late as up to 12 weeks’ post-childbirth. We note that this study population assessed women who had accessed care at a healthcare facility for routine antenatal or postnatal and was not able to assess the burden of maternal morbidity in women who did not access care and/or in women who may have experienced adverse pregnancy outcome (eg, stillbirth) and who do not generally attend for postnatal care. Such women might have an even higher burden of pregnancy-related morbidity. However, we note that many women in these settings do access care at least once during pregnancy (77%) and that skilled birth attendance during childbirth is more common (55%).^21 22^ We assessed women in two antenatal contacts only (early and late pregnancy) and future studies could consider assessing women in each trimester. We note that the study population we assessed in each country may not be generalisable to different regions of the same country or to other LMICs.

### Interpretation

This paper describes the burden of maternal morbidity at five different stages during and after pregnancy in four LMIC settings. The overall burden of maternal morbidity and associated factors have been described previously by this research group.^6^ At present, it is challenging to interpret measurements of maternal morbidity as there are few studies that have assessed maternal morbidity at different stages during and after pregnancy in LMIC settings^4^,^713 23^; and terms such as ‘maternal morbidity’, ‘maternal comorbidity’, ‘maternal multimorbidity’ have been used interchangeably. We consider our findings for each type of morbidity to studies that have explored maternal morbidity.

### Infectious morbidity (considering other evidence)

Infectious morbidity is traditionally considered to be a concern after childbirth as ‘puerperal sepsis’.^24^ However, our study demonstrates that women have early signs of infection (using an adapted SIRS score) from the first half of pregnancy (21.0%), and throughout later pregnancy and childbirth (25.1%–21.3%) and up to 12 weeks postnatal (23.1%); and that the potential causes of infection was not commonly HIV, tuberculosis or malaria. Our findings reflect a similar study that explored non-life threatening maternal morbidity where ‘fever of unknown origin’ was noted to be highest in the second half of pregnancy in Malawi (3.5%) and in the first half of pregnancy in Pakistan (3.6%).^7^ In another study, 6% of women reported febrile symptoms at 4–12 weeks; 11% at 12–24 weeks and 13% at 24–26 weeks after childbirth in Kenya.^23^ Further studies are needed to explore the significance of possible proxy measurements of infectious morbidity and to diagnose and manage underlying infection. In our study the prevalence of malaria and syphilis was highest in the first half of pregnancy stage, suggesting that with screening these conditions are detected and treated early; and/or prophylactic measures are successfully implemented (eg, malaria nets and anti-malaria prophylaxis). These findings are like those reported in a similar study where the highest percentage of women with malaria was in the early pregnancy stage in Malawi (8.8%) and Pakistan (2.1%).^7^ In our study, detection of HIV was highest in the early postnatal stage (5.4%). This is a similar finding to that of the Zafar *et al* study where the highest percentage of women diagnosed with HIV was in the postnatal stage in Malawi (16.5%) and Pakistan (7.0%).^7^ A suggested explanation for these findings could be that these women did not attend for and/or receive screening during antenatal care but did attend for childbirth at the healthcare facility and were screened for HIV as an inpatient.

### Medical and obstetrical morbidity (considering other evidence)

In our study, anaemia continues to be a major morbidity at all stages during and after pregnancy, and many women start their pregnancy anaemic. The severity of anaemia increased as the pregnancy continued, and more women had severe anaemia in the early postnatal stage; and this trend was similar in the four LMIC settings. In a study assessing maternal morbidity in Kenya, the highest percentage of women with anaemia (61.0%) was at 12–24 weeks after childbirth.^23^ However, this study did not assess women during pregnancy. In our study, the percentage of women with hypertension/pre-eclampsia was highest in the second half of pregnancy stage; and the highest percentage of women diagnosed with antepartum haemorrhage was in the first half of pregnancy stage. In our study, the highest percentage of women reporting urinary incontinence was in the second half of pregnancy stage 6.6%. In a similar study, the highest percentage of women reporting incontinence (urine or faeces) was in the postnatal stage in Malawi (0.9%) and Pakistan (4.7%).^7^ In the Cherish *et al* study, 2% of women had urine incontinence at 12–24 weeks, and 1% of women had urine incontinence at 4–12 weeks and 24–26 weeks after childbirth in Kenya.^23^ In our study 3.3% of women continued to report urinary incontinence in the late postnatal stage and this may represent possible early obstetric fistula. However, in our study women who reported persistent urinary incontinence postnatally were referred for specialist input and we did not have capacity to follow these patients up for a confirmed diagnosis of possible obstetric fistula.

### Psychological morbidity (considering other evidence)

Psychological morbidity is traditionally considered to be more significant after pregnancy, often termed ‘postnatal depression’. The findings of the prevalence of psychological morbidity in our study are higher than previous global estimates of 10% of women during pregnancy and 13% of women after childbirth being affected by psychological ill-health.^25^ The findings from our study are more like those of a systematic review of studies from LMIC settings where psychological morbidity was reported to affect 15.6% of women during pregnancy and 19.8% after pregnancy.^26^ Another review reported a prevalence of one in four women in LMIC settings reporting depression during pregnancy and one in five reported depression after pregnancy.^27^ The authors of the review suggest that the figures in LMIC settings are twice the rate of women in high-income countries and suggest that psychological morbidity in general is not reported, not assessed properly, infrequently recognised and under-treated in many LMIC.^26^

### Social morbidity (considering other evidence)

In our study, high percentages of women reported domestic violence and low percentages of women reported substance misuse. In a similar study, 12.8% of women self‐reported exposure to violence during pregnancy and 11.0% after childbirth.^1^ In a study exploring maternal morbidity in Kenya, only postnatal women were assessed, and more women reported domestic violence—physical (23.3%), sexual (56.9%)—and substance misuse (11%) at 12–24 weeks compared with 4–12 weeks and 24–26 weeks after childbirth in Kenya.^23^

### Practical and research recommendations

With emerging evidence of the different types of physical multimorbidity and comorbidity at specific stages during pregnancy and after childbirth, there is a need for comprehensive and detailed longitudinal studies of women from early pregnancy to an extended postpartum period to understand how health and symptoms and signs of ill-health change over time and how current antenatal and postnatal programmes can be improved to address these.^28^ There is ample evidence that psychological and social morbidity are associated with adverse consequences for the mother and the baby, both short and long-term,^29^,^33^ and there is a need to further explore and document how these types of maternal morbidities are interlinked and associated. There is a need to refine the current definition of maternal morbidity considering the conceptualisations of ‘maternal multimorbidity’ and/or ‘maternal comorbidities’, and/or related constructs; and to clarify the timeframe over which maternal morbidity impacts a woman’s health and well-being.^14^ Similarly, there is a gap between measuring morbidity for programmatic purposes and assessing its actual impact on a woman’s sense of well-being.^34^ Recent studies have explored women’s lived experiences of ill-health and perceptions of their health needs during and after pregnancy and these findings, in addition to the findings of our study, need to be considered when developing maternal health interventions to improve the quality of maternity care at different stages during pregnancy and after childbirth for women living in LMIC.^35 36^

## Conclusion

The overall findings from our study suggest that there is a large burden of physical morbidity, (including infectious, medical, obstetrical), psychological and social comorbidities in women during pregnancy and up to 12 weeks postnatal who access care in different levels of healthcare facilities across the four LMIC in which this study was conducted. Most importantly, the overall burden of ill-health is not simply at one ‘high risk’ stage of pregnancy, but women report and/or are diagnosed with significant physical morbidity and psychological and social comorbidities throughout the continuum of pregnancy and childbirth. At present, available antenatal and especially postnatal care packages across different settings are not adequate to screen for all forms of physical morbidity (infectious, medical, obstetrical) and psychological and social comorbidities; and do not address the needs of women in a comprehensive holistic way. Anaemia and possible signs of early infection represent a significant burden of ill-health along the continuum of pregnancy and childbirth and may be useful clinical proxy markers for physical maternal morbidity. There is a need to increase the focus of high quality comprehensive maternity care (including mental and social healthcare screening and management), from the first half of pregnancy through to the late postnatal stage, to ensure improved health and well-being for women and their babies during and after pregnancy, and not just at the time of childbirth. There is a need for further research to understand how to support healthcare providers to screen for and provide evidence based care all aspects of maternal multimorbidity (physical, psychological and social) at all contacts during pregnancy and after childbirth.

## Supplementary material

10.1136/bmjopen-2021-050287 online supplemental file 1

## Data Availability

Data are available upon reasonable request.
