# Supplementary material for: Physical morbidity and psychological and social comorbidities at five stages during pregnancy and after childbirth: a multicountry cross-sectional survey
Source: BMJ Open. 2022 Apr 25;12(4):e050287. doi: 10.1136/bmjopen-2021-050287 (PMC9039410; doi:10.1136/bmjopen-2021-050287)
Supplement: online supplemental file 1 [file bmjopen-2021-050287supp001.pdf]

SUPPLEMENTARY TABLES

Supplementary Table 1: Number of women per country, per assessment stage and per healthcare facility level (n=11,454)

Supplementary Table 2: Infectious, medical, and obstetric morbidity identified per assessment stage for women living in India (n=2,099)

Supplementary Table 3: Infectious, medical, and obstetric morbidity identified per assessment stage for women living in Pakistan (n=3,287)

Supplementary Table 4: Infectious, medical, and obstetric morbidity identified per assessment stage for women living in Kenya (n=3,145)

Supplementary Table 5: Infectious, medical, and obstetric morbidity identified per assessment stage for women living in Malawi (n=2,923)

Supplementary Table 6: Psychological and social morbidity per assessment stage for women living in India (n=2,099)

Supplementary Table 7: Psychological and social morbidity of women per assessment stage for women living in for all countries combined for Pakistan (n= 3,287)

Supplementary Table 8: Psychological and social morbidity per assessment stage for women living in Kenya (n=3,145)

Supplementary Table 9: Psychological and social morbidity per assessment stage for women living in Malawi (n= 2,923)

Supplementary Table 1: Socio-demographic and obstetric characteristics of women by stage and for all stages combined (n=11,454)

Assessment stage	First half of pregnancy	Second half of pregnancy	Within 24 hours of childbirth	Early postnatal	Late postnatal	Total	P value
**Number of women**	**2,204**	**2,425**	**2,250**	**2,264**	**2,311**	**11,454**	
	**%**	**%**	**%**	**%**	**%**	**%**	
**Age category (years)**							
**<20**	11.0	9.4	11.2	11.3	11.3	10.8	0.007
**20-24**	28.8	26.8	30.6	31.7	28.1	29.2	
**25-29**	30.7	29.2	29.4	29.8	30.9	30.0	
**30-34**	15.7	17.8	16.4	15.1	16.4	16.3	
**≥35**	8.0	8.7	6.6	7.0	7.2	7.5	
**Parity**							
**0 or 1**	34.8	30.3	35.7	38.6	35.0	34.8	<0.001
**2-4**	54.7	56.9	50.8	51.1	55.6	53.8	
**≥5**	5.9	8.5	6.1	5.7	5.8	6.4	
**Marital status**							
**Single**	6.2	6.1	4.9	5.7	4.5	5.5	0.16
**Married**	92.7	92.5	94.1	93.3	93.7	93.3	
**Socioeconomic status (SES)**							
**1st quintile (upper)**	13.7	10.4	10.8	11.2	11.2	11.4	<0.001
**2nd quintile**	16.5	15.8	15.4	17.4	16.7	16.3	
**3rd quintile**	31.0	28.8	30.1	28.5	31.8	30.0	
**4th quintile**	27.5	28.7	29.4	29.3	27.4	28.4	
**5th quintile (lower)**	8.7	12.1	11.7	10.6	10.3	10.7	
**Education level completed**							
**None**	17.2	18.0	20.4	20.9	19.3	19.2	<0.001
**Primary**	33.1	34.0	35.9	34.1	34.0	34.2	
**Secondary**	30.0	26.6	25.7	28.1	26.7	27.4	
**Post-secondary**	15.9	15.6	13.1	13.7	15.5	14.8	

<u>Supplementary Table 2:</u> Infectious, medical, and obstetric morbidity identified per assessment stage for women living in India (n=2,099)

**Assessment stage**	**First half of pregnancy**	**Second half of pregnancy**	**Within 24 hours of childbirth**	**Early postnatal**	**Late postnatal**	**Total**
Number of women[a]*	416	397	423	432	431	2099
	**n (%)**	**n (%)**	**n (%)**	**n (%)**	**n (%)**	**n (%)**
**INFECTIOUS MORBIDITY**						
**Condition**						
HIV	2 (0.5%)	2 (0.5%)	1 (0.2%)	1 (0.2%)	0	6 (0.3%)
Malaria	0	0	0	0	2 (0.5%)	2 (0.1%)
Syphilis	0	0	0	0	0	0
Positive screening for chest infection/ possible TB	2 (0.5%)	1 (0.2%)	1 (0.2%)	2 (0.5%)	2 (0.5%)	8 (0.4%)
Septic Inflammatory Response Syndrome (SIRS)[b]	43 (10.3%)	66 (16.6%)	47 (11.1%)	63 (14.6%)	70 (16.2%)	289 (13.8%)
**MEDICAL OR OBSTETRIC MORBIDITY**						
**Condition**						
Anaemia[c]	184 (44.2%)	228 (57.4%)	282 (66.7%)	309 (71.5%)	265 (61.5%)	1268 (60.4%)
Severe anaemia	4 (1.0%)	13 (3.3%)	12 (2.8%)	17 (3.9%)	5 (1.2%)	51 (2.4%)
Hypertension	2 (0.5%)	16 (4.0%)	7(1.6%)	7 (1.6%)	1 (0.2%)	33 (1.6%)
Low BMI (<18.5 kg/m^2)	30 (7.4%)	9 (2.3%)	9 (2.1%)	9 (2.1%)	10 (2.3%)	67 (3.2%)
High BMI (> 30kg/m^2)	55 (13.6%)	153 (38.9%)	109 (26.0%)	97 (22.5%)	107 (25%)	521 (25.1)
Pre-eclampsia	n/a	0	0	1 (0.2%)	n/a	1 (0.1%)
Urine incontinence	13 (3.1%)	21 (5.3%)	0	2 (0.5%)	0	36 (1.7%)
Antenatal haemorrhage	0	0	n/a	n/a	n/a	0
**At least 1 medical or obstetric condition**	202 (48.6%)	254 (64.0%)	285 (67.4%)	311 (72.0%)	266 (61.7%)	1318 (62.8%)

[a] Where data were missing for a condition the condition was regarding as being absent for purposes of deriving morbidities

[b] CRP was not measured at some primary level facilities in Malawi and Pakistan. Only participants for whom a CRP result was obtained are included in these statistics.

[c]Anaemia is defined as Hb< 11.0g/dL and severe anaemia is defined as Hb<7g/dL

Supplementary Table 3: **Infectious, medical, and obstetric morbidity identified per assessment stage for women living in Pakistan (n=3,287)**

**Assessment stage**	**First half of pregnancy**	**Second half of pregnancy**	**Within 24 hours of childbirth**	**Early postnatal**	**Late postnatal**	**Total**
Number of women[a]*	607	768	654	618	640	3287
	**n (%)**	**n (%)**	**n (%)**	**n (%)**	**n (%)**	**n (%)**
**INFECTIOUS MORBIDITY**						
**Condition**						
HIV	0	1 (0.1%)	7 (1.1%)	2 (0.3%)	1 (0.2%)	11 (0.3%)
Malaria	0	0	0	1 (0.2%)	0	1 (0.03%)
Syphilis	0	0	0	0	0	0
Positive screening for chest infection/ possible TB	7 (1.1%)	11 (1.4%)	2 (0.3%)	2 (0.3%)	3 (0.5%)	25 (0.8%)
Septic Inflammatory Response Syndrome (SIRS)[b]	67 (11.0%)	122 (15.9%)	54 (8.3%)	70 (11.3%)	55 (8.6%)	368 (11.2%)
**MEDICAL OR OBSTETRIC MORBIDITY**						
**Condition**						
Anaemia[c]	389 (64.1%)	590 (76.8%)	435 (66.5%)	437 (70.7%)	375 (58.6%)	2226 (67.7%)
Severe anaemia	9 (1.5%)	27 (3.5%)	18 (2.8%)	22 (3.6%)	5 (0.8%)	81 (2.5%)
Body mass index ≤18.5 kg/m^2	37 (6.2%)	16(2.2%)	7(1.1%)	11 (1.8%)	10 (1.6%)	81 (2.5%)
High BMI (> 30kg/m^2)	289 (48.5%)	470 (63.2%)	306 (48.6%)	325 (53.7%)	307 (49.8%)	1697 (53.2%)
Hypertension	18 (3.0%)	67 (8.7%)	54 (8.3%)	40 (6.5%)	27 (4.2%)	206 (6.3%)
Pre-eclampsia	n/a	26 (3.4%)	8 (1.2%)	6 (1.0%)	n/a	40 (2.0%)
Urine incontinence	40 (6.6%)	106 (13.8%)	64 (9.8%)	39 (6.3%)	65 (10.2%)	314 (9.5%)
Antenatal haemorrhage	80 (13.2%)	60 (7.8%)	n/a	n/a	n/a	140 (10.2%)
**At least 1 medical or obstetric condition**	422 (69.5%)	619 (80.6%)	455 (69.6%)	455 (73.6%)	388 (60.6%)	2339 (71.2%)

[a] Where data were missing for a condition the condition was regarding as being absent for purposes of deriving morbidities

[b] CRP was not measured at some primary level facilities in Malawi and Pakistan. Only participants for whom a CRP result was obtained are included in these statistics.

[c] Anaemia is defined as Hb< 11.0g/dL and severe anaemia is defined as Hb<7g/dL

Supplementary Table 4: **Infectious, medical, and obstetric morbidity identified per assessment stage for women living in Kenya (n=3,145)**

**Assessment stage**	**First half of pregnancy**	**Second half of pregnancy**	**Within 24 hours of childbirth**	**Early postnatal**	**Late postnatal**	**Total**

Number of women[a]*	592	684	592	620	657	3145
	**n (%)**	**n (%)**	**n (%)**	**n (%)**	**n (%)**	**n (%)**
**INFECTIOUS MORBIDITY**						
**Condition**						
HIV	24 (4.0%)	19 (2.8%)	24 (4.0%)	25 (4.0%)	25 (3.8%)	117 (3.7%)
Malaria	2 (0.3%)	2 (0.3%)	1 (0.2%)	2 (0.3%)	0	7 (0.2%)
Syphilis	2 (0.3%)	2 (0.3%)	1 (0.2%)	0	3 (0.5%)	8 (0.2%)
Positive screening for chest infection/ possible TB	3 (0.5%)	6 (0.9%)	5 (0.8%)	3 (0.5%)	5 (0.8%)	22 (0.7%)
Septic Inflammatory Response Syndrome (SIRS)[b]	206 (34.8%)	267 (39.0%)	197 (33.3%)	278 (44.8%)	199 (30.3%)	1147 (36.5%)
**MEDICAL OR OBSTETRIC MORBIDITY**						
**Condition**						
Anaemia[c]	102 (17.2%)	163 (23.8%)	146 (24.7%)	204 (32.9%)	130 (19.8%)	745 (23.7%)
Severe anaemia	7 (1.2%)	4 (0.6%)	7 (1.2%)	12 (1.9%)	12 (1.8%)	65 (2.1%)
Body mass index ≤18.5 kg/m^2	18 (3.1%)	8 (1.2%)	10 (1.7%)	7 (1.2%)	3 (0.5%)	46 (1.5%)
High BMI (> $30kg/m^2$)	278 (47.7%)	404 (59.8%)	328 (56.2%)	350 (57.6%)	325 (50.3%)	1685 (54.4%)
Hypertension	7 (1.2%)	19 (2.8%)	13 (2.2%)	16 (2.6%)	15 (2.3%)	70 (2·2%)
Pre-eclampsia	n/a	8 (1.2%)	0	4 (0.6%)	n/a	12 (0.6%)
Urine incontinence	12 (2.0%)	23 (3.4%)	2 (0.3%)	3 (0.5%)	9 (1.4%)	49 (1.6%)
Antenatal haemorrhage	54 (9.1%)	12 (1.7%)	n/a	n/a	n/a	66 (5.2%)
**At least 1 medical or obstetric condition**	142 (24.0%)	197 (28.8%)	156 (26.3%)	214 (34.5%)	143 (21.8%)	852 (27.1%)

[a] Where data were missing for a condition the condition was regarding as being absent for purposes of deriving morbidities

[b] CRP was not measured at some primary level facilities in Malawi and Pakistan. Only participants for whom a CRP result was obtained are included in these statistics.

[c]Anaemia is defined as Hb< 11.0g/dL and severe anaemia is defined as Hb<7g/dL

Supplementary Table 5: Infectious, medical, and obstetric morbidity identified per assessment stage for women living in Malawi (n=2923)

Assessment stage	First half of pregnancy	Second half of pregnancy	Within 24 hours of childbirth	Early postnatal	Late postnatal	Total
Number of women [a]	589	576	581	594	583	2923
	**n (%)**	**n (%)**	**n (%)**	**n (%)**	**n (%)**	**n (%)**
**INFECTIOUS MORBIDITY**						
HIV	73 (12.4%)	85 (14.8%)	75 (12.9%)	95(16.0%)	90(15.4%)	418 (14.3%)
Malaria	83 (14.1%)	71 (12.3%)	58 (10.0%)	40 (6.7%)	47 (8.1%)	299 (10.2%)
Syphilis	34 (5.8%)	22 (3.8%)	18 (3.1%)	16 (2.7%)	9 (1.5%)	99 (3.4%)
Positive screening for chest infection/ possible TB	6 (1.0%)	6 (1.0%)	2 (0.3%)	5 (0.8%)	0	19 (0.7%)
Septic Inflammatory Response Syndrome (SIRS)[b]	164 (27.8%)	154 26.7(%)	174 (30.0%)	179 (30.1%)	168 (28.8%)	839 (28.7%)
**MEDICAL OR OBSTETRIC MORBIDITY**						
Anaemia[c]	225 (38.2%)	245 (42.5%)	233 (40.1%)	263 (44.3%)	215 (36.9%)	1181 (40.4%)
Severe anaemia	8 (1.4%)	3 (0.5%)	4 (0.7%)	12 (2.0%)	6 (1.0%)	33 (1.1%)
Body mass index ≤18.5 kg/m^2	18 (3.1%)	7 (1.2%)	19 (3.3%)	22 (3.7%)	22 (3.8%)	88 (3.0%)
High BMI (> 30kg/m^2)	164 (28.3%)	237 (41.6%)	169 (29.2%)	186 (31.5%)	208 (36.1%)	964 (33.3%)
Hypertension	6 (1.0%)	9 (1.6%)	8 (1.4%)	3 (0.5%)	5(0.9%)	31 (1.1%)
Pre-eclampsia	n/a	17 (2.9%)	10 (1.7%)	6 (1.0%)	n/a	49 (2.8%)
Urine incontinence	2 (0.3%)	11 (1.9%)	3 (0.5%)	0	2 (0.3%)	18 (0.6%)
Antenatal haemorrhage	5 (0.9%)	2 (0.4%)	n/a	n/a	n/a	7 (0.6%)
**At least one medical or obstetric condition**	232 (39.4%)	258 (44.8%)	239 (41.1%)	265 (44.6%)	220 (37.7%)	1214 (41.5%)

[a] Where data were missing for a condition the condition was regarding as being absent for purposes of deriving morbidities

[b] CRP was not measured at some primary level facilities in Malawi and Pakistan. Only participants for whom a CRP result was obtained are included in these statistics.

[c] Anaemia is defined as Hb< 11.0g/dL and severe anaemia is defined as Hb<7g/dL

Supplementary Table 6: **Psychological and social morbidity per assessment stage for women living in India (number of women assessed n=2099)**

stage		First half of pregnancy	Second half of pregnancy	Within 24 hours of childbirth	Early postnatal	Late postnatal	Total
Number of women*		416	397	423	432	431	2099
		n %	n %	n %	n %	n %	n %
**PSYCHOLOGICAL MORBIDITY**							
**EDPS ≥ 10**		45 (10.8%)	103 (25.9%)	86 (20.3%)	86 (19.9%)	84 (19.5%)	404 (19.2%)
**Thoughts of self-harm**		40 (9.6%)	79 (19.9%)	72 (17.0%)	71 (16.4%)	66 (15.3%)	328 (15.6%)
**EDPS ≥ 10 and/or thoughts of self-harm**		60 (14.4%)	116 (29.2%)	102 (24.1%)	99 (22.9%)	95 (22.0%)	472 (22.5%)
**SOCIAL MORBIDITY**							
**Domestic violence**							
**HITS score >4**	Husband and/or family	159 (38.2%)	173 (43.6%)	153 (36.2%)	189 (43.7%)	159 (36.9%)	833 (39.7%)
	Husband	154 (37.0%)	164 (41.3%)	152 (35.9%)	185 (42.8%)	155 (36.0%)	810 (38.6%)
	Family	35 (8.4%)	67 (16.9%)	28 (6.6%)	52 (12.0%)	29 (6.7%)	211 (10.0%)
**HITS score >10**	Husband and/or family	23 (5.5%)	36 (9.1%)	18 (4.3%)	33 (7.6%)	17 (3.9%)	127 (6.0%)
	Husband	19 (4.6%)	27 (6.8%)	16 (3.8%)	30 (6.9%)	15 (3.5%)	107 (5.1%)
	Family	8 (1.9%)	22 (5.5%)	3 (0.7%)	15 (3.5%)	8 (1.9%)	56 (2.7%)
**Substance misuse**							
**Use of alcohol, sedatives, inhalants, or tobacco in last 3 months**		6 (1.4%)	9 (2.3%)	4 (0.9%)	12 (2.8%)	5 (1.2%)	36 (1.7%)
**Intervention required**		4 (1.0%)	7 (1.8%)	0	5 (1.2%)	1 (0.2%)	17 (0.8%)

ASSIST: Alcohol, Smoking and Substance Involvement Screening Test; EPDS: Edinburgh Postnatal Depression Scale; HITS: Hurt, Insulted, Threatened, Screamed at.

Supplementary Table 7: Psychological and social morbidity of women per assessment stage for women living in for all countries combined for Pakistan (number of women assessed n= 3287)

stage		First half of pregnancy	Second half of pregnancy	Within 24 hours of childbirth	Early postnatal	Late postnatal	Total
Number of women*		607	768	654	618	640	3287
		n %	n %	n %	n %	n %	n %
**PSYCHOLOGICAL MORBIDITY**							
**EDPS ≥ 10**		277 (45.6%)	349 (45.4%)	243 (37.2%)	256 (41.4%)	243 (38.0%)	1368 (41.6%)
**Thoughts of self-harm**		130 (21.4%)	242 (31.5%)	194 (29.7%)	200 (32.4%)	212 (33.1%)	978 (29.7%)
**EDPS ≥ 10 and/or thoughts of self-harm**		297 (48.9%)	379 (49.3%)	323 (49.4%)	308 (49.8%)	315 (49.2%)	1622 (49.3%)
**SOCIAL MORBIDITY**							
**Domestic violence**							
**HITS score >4**	Husband and/or family	308 (50.7%)	464 (60.4%)	371 (56.7%)	382 (61.8%)	314 (49.1%)	1839 (55.9%)
	Husband	183 (30.1%)	340 (44.3%)	255 (39.0%)	255 (41.3%)	195 (30.5%)	1228 (37.4%)
	Family	197 (32.4%)	246 (32.0%)	217 (33.2%)	199 (32.2%)	184 (28.7%)	1043 (31.7%)
**HITS score >10**	Husband and/or family	109 (18.0%)	185 (24.1%)	103 (15.7%)	115 (18.6%)	104 (16.2%)	616 (18.7%)
	Husband	64 (10.5%)	150 (19.5%)	59 (9.0%)	70 (11.3%)	68 (10.6%)	411 (12.5%)
	Family	50 (8.2%)	47 (6.1%)	55 (8.4%)	47 (7.6%)	47 (7.3%)	246 (7.5%)
**Substance misuse**							
**Use of alcohol, sedatives, inhalants, or tobacco in last 3 months**		61 (10.0%)	53 (6.9%)	45 (6.9%)	74 (12.0%)	60 (9.4%)	293 (8.9%)
**Intervention required**		7 (1.1%)	13 (1.7%)	5 (0.8%)	12 (1.9%)	20 (3.1%)	57 (1.7%)

ASSIST: Alcohol, Smoking and Substance Involvement Screening Test; EPDS: Edinburgh Postnatal Depression Scale; HITS: Hurt, Insulted, Threatened, Screamed at.

Supplementary Table 8: Psychological and social morbidity per assessment stage for women living in Kenya (n=3145)

Assessment stage		First half of pregnancy	Second half of pregnancy	Within 24 hours of childbirth	Early postnatal	Late postnatal	Total
Number of women*		592	684	592	620	657	3145
		**n %**	**n %**	**n %**	**n %**	**n %**	**n %**
**PSYCHOLOGICAL MORBIDITY**							
**EDPS ≥ 10**		86 (14.5%)	101 (14.8%)	81 (13.7%)	72 (11.6%)	49 (7.5%)	389 (12.4%)
**Thoughts of self-harm**		28 (4.7%)	47 (6.9%)	29 (4.9%)	20 (3.2%)	19 (2.9%)	143 (4.5%)
**EDPS ≥ 10 and/or thoughts of self-harm**		94 (15.9%)	126 (18.4%)	95 (16.0%)	84 (13.5%)	60 (9.1%)	**459 (14.6%)**
**SOCIAL MORBIDITY**							
**Domestic violence**							
**HITS score >4**	Husband and/or family	99 (16.7%)	201 (29.4%)	130 (22.0%)	105 (17.0%)	144 (21.9%)	679 (21.6%)
	Husband	78 (13.2%)	176 (25.7%)	108 (18.2%)	88 (14.2%)	125 (19.0%)	575 (18.3%)
	Family	29 (4.9%)	51 (7.5%)	35 (5.9%)	24 (3.9%)	47 (7.1%)	186 (5.9%)
**HITS score >10**	Husband and/or family	9 (1.5%)	26 (3.8%)	22 (3.7%)	12 (1.9%)	17 (2.6%)	86 (2.7%)
	Husband	8 (1.3%)	24 (3.5%)	18 (3.0%)	10 (1.6%)	15 (2.3%)	75 (2.4%)
	Family	2 (0.3%)	2 (0.3%)	6 (1.0%)	2 (0.3%)	2 (0.3%)	14 (0.4%)
**Substance misuse**							
**Use of alcohol, sedatives, inhalants, or tobacco in last 3 months**		40 (6.8%)	27 (3.9%)	42 (7.1%)	37 (6.0%)	63 (9.6%)	209 (6.6%)
**Intervention required**		9 (1.5%)	6 (0.9%)	9 (1.5%)	8 (1.3%)	14 (2.1%)	46 (1.5%)

ASSIST: Alcohol, Smoking and Substance Involvement Screening Test; EPDS: Edinburgh Postnatal Depression Scale; HITS: Hurt, Insulted, Threatened, Screamed at.

Supplementary Table 9: Psychological and social morbidity per assessment stage for women living in Malawi (number of women assessed n= 2923)

Assessment stage		First half of pregnancy	Second half of pregnancy	Within 24 hours of childbirth	Early postnatal	Late postnatal	Total
Number of women*		589	576	581	594	583	2923
		n %	n %	n %	n %	n %	n %
**PSYCHOLOGICAL MORBIDITY**							
**EDPS ≥ 10**		86 (14.6%)	88 (15.3%)	101 (17.4%)	89 (15.0%)	93 (15.9%)	457 (15.6%)
**Thoughts of self-harm**		48 (8.2%)	58 (10.1%)	64 (11.0%)	60 (10.1%)	65 (11.1%)	295 (10.1%)
**EDPS ≥ 10 and/or thoughts of self-harm**		91 (15.4%)	100 (17.4%)	110 (18.9%)	100 (16.8%)	105 (18.0%)	506 (17.3%)
**SOCIAL MORBIDITY**							
**Domestic violence**							
**HITS score >4**	Husband and/or family	91 (15.4%)	140 (24.3%)	102 (17.6%)	94 (15.8%)	105 (18.0%)	532 (18.2%)
	Husband	60 (10.2%)	96 (16.7%)	82 (14.1%)	74 (12.5%)	85 (14.6%)	397 (13.6%)
	Family	67 (11.4%)	80 (13.9%)	78 (13.4%)	66 (11.1%)	74 (12.7%)	365 (12.5%)
**HITS score >10**	Husband and/or family	20 (3.4%)	29 (5.0%)	29 (5.0%)	27 (4.5%)	32 (5.5%)	137 (4.7%)
	Husband	10 (1.7%)	22 (3.8%)	16 (2.7%)	17 (2.9%)	23 (3.9%)	88 (3.0%)
	Family	14 (2.4%)	9 (1.6%)	21 (3.6%)	15 (2.5%)	16 (2.7%)	75 (2.6%)
**Substance misuse**							
**Use of alcohol, sedatives, inhalants, or tobacco in last 3 months**		28 (4.7%)	23 (4.0%)	31 (5.3%)	23 (3.9%)	29 (5.0%)	134 (4.6%)
**Intervention required**		18 (3.1%)	12 (2.1%)	20 (3.4%)	17 (2.9%)	15 (2.6%)	82 (2.8%)

ASSIST: Alcohol, Smoking and Substance Involvement Screening Test; EPDS: Edinburgh Postnatal Depression Scale; HITS: Hurt, Insulted, Threatened, Screamed at.
