# Supplementary material for: Physical morbidity and psychological and social comorbidities at five stages during pregnancy and after childbirth: a multicountry cross-sectional survey
Source: BMJ Open. 2022 Apr 25;12(4):e050287. doi: 10.1136/bmjopen-2021-050287 (PMC9039410; doi:10.1136/bmjopen-2021-050287)
Supplement: Supplementary file 2 [file bmjopen-2021-050287.reviewer_comments.pdf]

## PEER REVIEW HISTORY

BMJ Open publishes all reviews undertaken for accepted manuscripts. Reviewers are asked to complete a checklist review form (**http://bmjopen.bmj.com/site/about/resources/checklist.pdf**) and are provided with free text boxes to elaborate on their assessment. These free text comments are reproduced below.

## ARTICLE DETAILS

| | |
|---|---|
| **TITLE (PROVISIONAL)** | PHYSICAL MORBIDITY AND PSYCHOLOGICAL AND SOCIAL CO-MORBIDITIES AT FIVE STAGES DURING PREGNANCY AND AFTER CHILDBIRTH – A MULTI-COUNTRY CROSS SECTIONAL SURVEY |
| **AUTHORS** | McCauley, Mary; White, Sarah; Bar-Zeev, Sarah; Godia, Pamela; Mittal, Pratima; Zafar, Shamsa; van den Broek, Nynke |

## VERSION 1 – REVIEW

| | |
|---|---|
| **REVIEWER** | Sanchez-Polan, Miguel<br>Universidad Politecnica de Madrid |
| **REVIEW RETURNED** | 11-May-2021 |

| | |
|---|---|
| **GENERAL COMMENTS** | I appreciate the oportunity to review this article. I found a very carefully designed study, with a big sample size and a necessary issue to address.<br><br>However, I have a few comments to the authors (see below)<br><br>Specific comments:<br><br>Abstract:<br><br>Please, be more concise with the aim of the study at objective section.<br><br>I found a structured abstract without some necessary sections (desing and interventions). Please, provide them.<br><br>Please, be more concise with inclusion/exclusion criteria. Reading the article deeply, the authors said "Women who were too ill to participate... were excluded". Provide more detailed criteria.<br><br>Materials and methods:<br><br>I have a question from authors in this section, why did they only take data on two stages during pregnancy? It would have been interesting to measure data in each trimester of pregnancy.<br><br>Edinburgh Postnatal Depression Scale, it is not a questionnaire to assess psychological health, it was designed to measure depression and anxiety symptoms during and after pregnancy. Please change this sentence in page 7.<br><br>References: |

| | |
|---|---|
| | The reference formatting needs some improvement. With a random check is possible to see some inconsistencies such as missing dot after the journal name, or the year in a wrong position. |

| | |
|---|---|
| **REVIEWER** | Norhayati, Mohd Noor<br>University Sains Malaysia - Health Campus, Department of Family Medicine |
| **REVIEW RETURNED** | 19-Sep-2021 |

| | |
|---|---|
| **GENERAL COMMENTS** | Rationale<br>How would the five different stages contribute to clinical and public health importance when it involves multiple countries?<br><br>Definition of maternal morbidity. It is understood how physical morbidity contributes to medical outcomes. Psychological or social comorbidity might affect the wellbeing of a woman, but how would it, to an extent, affecting pregnancy outcome?<br><br>Results<br>The results were heavily written in the text. Do not repeat the information in tables.<br><br>Discussion<br>The Discussion section repeats the results.<br>The discussion is weak in that it lacks an explanation of the results.<br>Please organize the discussion.<br><br>Conclusion<br>The descriptive nature of the study limits the application of the study findings. |

**VERSION 1 – AUTHOR RESPONSE**

Reviewer: 1
Dr. Miguel Sanchez-Polan, Universidad Politecnica de Madrid

Comments to the Author:
I appreciate the opportunity to review this article. I found a very carefully designed study, with a big sample size and a necessary issue to address.

Thank you very much for these positive comments.

However, I have a few comments to the authors (see below)

Please, be more concise with the aim of the study at objective section.

Thank you. Text at the end of the Introduction section has been amended to be clearer.

I found a structured abstract without some necessary sections (design and interventions). Please, provide them.

Thank you, we used the abstract format as requested by the journal. However, we have checked this again.

The design was an observational cross-sectional study, already mentioned in the abstract.

There was no intervention as part of this study design but women in which any ill-health was detected were then given care or referral as necessary as part of the antenatal and /or postnatal care package in place.

Please, be more concise with inclusion/exclusion criteria. Reading the article deeply, the authors said "Women who were too ill to participate... were excluded". Provide more detailed criteria.

Thank you. Inclusion criteria included: all women attending for routine care at the study healthcare facilities. Exclusion criteria were those women who were inpatients and severely unwell (and therefore physically unable to speak to complete the health interview and examination) for example women with an altered conscious level due to a massive obstetric haemorrhage or eclamptic fit and were in a high dependency unit or intensive care setting. We have further clarified this in the methods section.

Materials and methods:

I have a question from authors in this section, why did they only take data on two stages during pregnancy? It would have been interesting to measure data in each trimester of pregnancy.

Thank you for this comment. We appreciate that traditionally antenatal care is categorised across three trimesters. We used two assessment points during pregnancy as in the settings in which this study was performed across four low-and middle-income countries, women often do not present to the healthcare facilities during the first trimester (first 12 weeks), tending to book later compared to women in high-income countries. We have now highlighted this in the Discussion section as a limitation in the section ‘Strengths and Limitations’.

Edinburgh Postnatal Depression Scale, it is not a questionnaire to assess psychological health. It was designed to measure depression and anxiety symptoms during and after pregnancy. Please change this sentence in page 7.

Thank you for this comment. We used the 10-question Edinburgh Postnatal Depression Scale (EPDS) in our study as this is a valuable and efficient way of identifying patients at risk for perinatal depression and is the most common validated data collection tool to assess psychological morbidity in many studies across different languages and settings.

As per original reference [1], the EPDS is easy to administer and has proven to be an effective screening tool for symptoms of depression. Mothers who score high scores are likely to be suffering from a depressive illness of varying severity.

As per original reference [1], we appreciate that the Edinburgh Postnatal Depression Scale was not designed to and does not assess all types of psychological health (for example, anxiety neuroses, phobias, psychosis, personality disorders).

We refer to the following references cited.

References

[1] Cox, J.L., Holden, J.M., and Sagovsky, R. 1987. Detection of postnatal depression: Development of the 10-item Edinburgh Postnatal Depression Scale. British Journal of Psychiatry 150:782-786.

References:

The reference formatting needs some improvement. With a random check is possible to see some inconsistencies such as missing dot after the journal name, or the year in a wrong position.

My sincere apologies. All the references have been checked and amended to ensure consistency.

Reviewer: 2
Prof. Mohd Noor Norhayati, University Sains Malaysia - Health Campus
Comments to the Author:

Rationale
How would the five different stages contribute to clinical and public health importance when it involves multiple countries?

Antenatal (ANC) and Postnatal Care (PNC) are key essential health services packages provided currently globally in all income setting and are considered key Public Health interventions during and after pregnancy for the mother and baby. In addition, ANC and PNC serve as ‘platforms’ for extended public health services and messaging as women are often the primary point of access to health services for the larger family.

This paper also highlights the overall burden of ill-health during and after pregnancy which is often ‘hidden’ and/or underestimated especially in low- and middle -income countries.

The objective of this paper is to describe the prevalence and types of maternal morbidity and co-morbidities for each of five different stages during and after pregnancy and to assess if there are differences in the trends of occurrence and types of physical morbidity, and psychological and social co-morbidity at different stages and in different settings; in order to improve current antenatal and postnatal programmes in such settings, and to inform when the optimal timing is for screening, prevention, and management of maternal morbidity. These points or stages were chosen as these are the times that women in ‘real life settings’ do access care at both primary and secondary levels of the health system and can provide evidence for programming of public health programmes.

In the strengths and limitations section of this paper, we further highlight that the study population we assessed in each country may not be generalizable to different regions of the same country or to other low-and middle-income countries.

Definition of maternal morbidity. It is understood how physical morbidity contributes to medical outcomes. Psychological or social comorbidity might affect the wellbeing of a woman, but how would it, to an extent, affecting pregnancy outcome?

Thank you for this comment.
Regarding the definition - we consistently adopted the current definition of maternal morbidity as agreed at WHO level and which includes physical as well as psychological and social morbidity. (See References 9 and 10 in our paper).

There is no doubt that psychological and social comorbidity have an impact on women’s health and wellbeing. There are also many studies to demonstrate and advocate that psychological and social

comorbidity does affect not only the wellbeing of a woman but contribute to and/or result in adverse pregnancy outcome.

Regarding social morbidity, domestic violence during pregnancy is associated with poor health outcomes for both the woman and her unborn baby including maternal injury or death; and pregnancy complications such as placental abruption, premature rupture of membranes, preterm labour, low birth weight and stillbirth. [1-4]. Domestic violence also has life-long negative implications for a woman's general health including physical (chronic pain, migraines) and psychological (anxiety, depression, post-traumatic stress disorder) consequences [5-6].

Furthermore, compromised maternal mental health is associated with adverse consequences for the mother and the baby, both short and long term [7-9].

We thank you for raising this additional important point and have expanded our Discussion and referencing accordingly (Section Practical and Research Recommendations).

Additional References Added:


1. Shrestha M, Shrestha S, Shrestha B. Domestic violence among antenatal attendees in a Kathmandu hospital and its associated factors: a cross-sectional study. BMC Pregn Childb. 2016;16:360. doi:10.1186/s12884-016-1166-7.

2. Zakar R, Nasrullah M, Zakar MZ, Ali H. (2016), The association of intimate partner violence with unintended pregnancy and pregnancy loss in Pakistan. Int J Gynaecol Obstet. 2016;133(1):26–31. doi:10.1016/j.ijgo.2015.09.009.

3. Fikree F, Jafarey S, Korejo R, Khan A, Durocher J. (2004), Pakistani obstetricians' recognition of and attitude towards domestic violence screening. Int J Gynaecol Obstet. 2004;87(1):59–65.

4. Nelson HD, Bougatsas C, Blazina I. Screening Women for Intimate Partner Violence: A systematic review to update U.S Preventative Risk Force Recommendations. Ann Intern Med. 2012;156(11):796–808.

5. Usta J, Antoun J, Ambuel B, Khawaja M. Involving the Healthcare System in Domestic Violence: What Women Want. Ann Fam Med. 2012;10(3):213–20. doi:10.137o/afm.1336

6. Ellsberg M, Jansen HA, Heise L, Watts CH, Garcia-Moreno C. Intimate Partner Violence and Women's Physical and Mental Health in WHO Multi-Country Study on Women's Health and Domestic Violence: An Observational Study. Lancet. 2008;371(9619):1165–72. doi:10.1016/S0140-6736(08)60522-x.

7. Ambuel B, Hamberger LK, Guse CE, Melzer-Lange M, Phelan MB, Kistner A. Healthcare can change from within. J Fam Violence. 2013;28(8):833–47.

8. Manikkam L, Burns JK. Antenatal depression and its risk factors: an urban prevalence study in KwaZulu-Natal. S Afr Med J. 2012;102(12):940–4.

9. Patel V, Rahman A, Jacob K, Hughes M. Effect of maternal mental health on infant growth in low income countries: new evidence from South Asia. Br Med J. 2004;328(7443):820.

Results
The results were heavily written in the text. Do not repeat the information in tables.

We appreciate that we provide a lot of new data. We tried to avoid any duplication while still making it easy for the reader to follow. The results section has been edited to ensure that information is not repeated in the text. We have edited the Results text further. We have tried to highlight the most significant findings in the text whilst still providing the full data in tables – including supplementary tables as per Journal instruction.

Discussion
The Discussion section repeats the results.
The discussion is weak in that it lacks an explanation of the results.
Please organize the discussion.

Thank you – We have further strengthened the Discussion section.

As generally recommended, we used a structured discussion format with subheadings. As recommended the Discussion starts with a short summary of only the most pertinent findings (Main Findings). We have edited this further to avoid repetition.

Given that the definition and scope of 'maternal morbidity' is comparatively new - and there are few papers on this subject currently - we do find it important to elaborate on the findings whilst trying to remain concise.

Conclusion
The descriptive nature of the study limits the application of the study findings.

Whilst we appreciate that this study is an observational cross-section survey, this study has provided new data to demonstrate that maternal morbidity represents a significant burden of ill-health across all stages of pregnancy and that public health measures and proposed improvements in the maternal health service in low-and middle-income countries should screen for such types of maternal morbidity and be equipped and designed to provide compassionate care and empathy and/or referral for women who need it.

We do highlight that the data are not generalisable in the section on Limitations and recommend further studies.
